# Hemodynamic Correlates of Late Systolic Flow Velocity Augmentation in the Carotid Artery

**DOI:** 10.1155/2013/920605

**Published:** 2013-11-14

**Authors:** Kevin S. Heffernan, Wesley K. Lefferts, Jacqueline A. Augustine

**Affiliations:** Department of Exercise Science, Syracuse University, Syracuse, NY 13244, USA

## Abstract

*Background.* The contour of the common carotid artery (CCA) blood flow velocity waveform changes with age; CCA flow velocity increases during late systole, and this may contribute to cerebrovascular disease. Late systolic flow velocity augmentation can be quantified using the flow augmentation index (FAIx). We examined hemodynamic correlates of FAIx to gain insight into determinants of CCA flow patterns. *Methods.* CCA Doppler ultrasound and wave intensity analysis (WIA) were used to assess regional hemodynamics in 18 young healthy men (age 22 ± 1 years). Forward waves (*W*_1_) and backward waves (negative area, NA) were measured and used to calculate the reflection index (NA/*W*_1_ = RIx). Additional parameters included *W*_2_ which is a forward travelling expansion/decompression wave of myocardial origin that produces suction, CCA single-point pulse wave velocity (PWV) as a measure of arterial stiffness, and CCA pressure augmentation index (AIx). *Results.* Primary correlates of FAIx included *W*_2_ (*r* = − 0.52, *P* < 0.05), _log_RIx (*r* = 0.56, *P* < 0.05), and AIx (*r* = 0.60, *P* < 0.05). FAIx was not associated with CCA stiffness (*P* > 0.05). *Conclusions.* FAIx is a complex ventricular-vascular coupling parameter that is associated with both increased expansion wave magnitude (increased suction from the left ventricle) and increased pressure from wave reflections.

## 1. Introduction

Characterization of the carotid flow waveform is important as its contour determines regional shear stress, a strong determinant of atherosclerosis, and its pulsatility contributes to cerebral damage [1–3]. The carotid flow velocity waveform in healthy human arteries is unidirectional (predominantly antegrade) and triphasic, exhibiting characteristic peaks in early systole, late systole, and early diastole [4–6]. Late systolic flow velocity increases with age and contributes to increased cerebrovascular risk [4, 7]. Carotid late systolic flow velocity augmentation may be quantified using the carotid flow augmentation index (FAIx) [4]. It has been suggested that pressure from wave reflections is a primary determinant of late systolic flow augmentation as there is a strong association between carotid FAIx and carotid pressure AIx [4]. Additional vascular and hemodynamic correlates of FAIx remain unexplored. 

An additional novel hemodynamic factor that may influence the contour of the carotid flow wave is related to myocardial properties. During late systole as the left ventricle (LV) begins to untwist and myocardial shortening rate is reduced, there is rapid decline in outflow momentum and LV pressure [8, 9]. This results in propagation of a forward travelling expansion wave (also known as a decompression wave or rarefaction wave) [9] which creates a suction effect that applies a “braking” action to the column of blood from behind and actively decelerates flow [10, 11]. Suction waves contribute to late systolic/early diastolic flow in the coronary circulation [12], the aorta/aortic valve closure [8, 13], and the femoral artery [14]. Previous studies have identified the existence of a late systolic forward travelling expansion wave in the carotid artery as well [15, 16]. Whether this expansion/suction wave is specifically associated with carotid flow velocity in late systole is unknown. 

The purpose of this study was to examine hemodynamic correlates of CCA FAIx in young healthy men. Using Doppler-ultrasound, wave intensity analysis (WIA), and wave separation analysis (WSA), we measured carotid artery flow velocity, carotid stiffness, wave reflections, and suction. We hypothesized that in addition to pressure from wave reflections, the contour of the carotid flow waveform would be associated with a forward travelling expansion/suction wave. A secondary purpose was to explore target organ damage correlates of FAIx. We hypothesized that, FAIx would be associated with CCA intima media thickness (a measure of subclinical atherosclerosis/vascular wall damage) [17–19] and cerebral pulsatility index (a known correlate of cerebral microvascular damage) [20–22]. 

## 2. Methods

**Figure 1:**
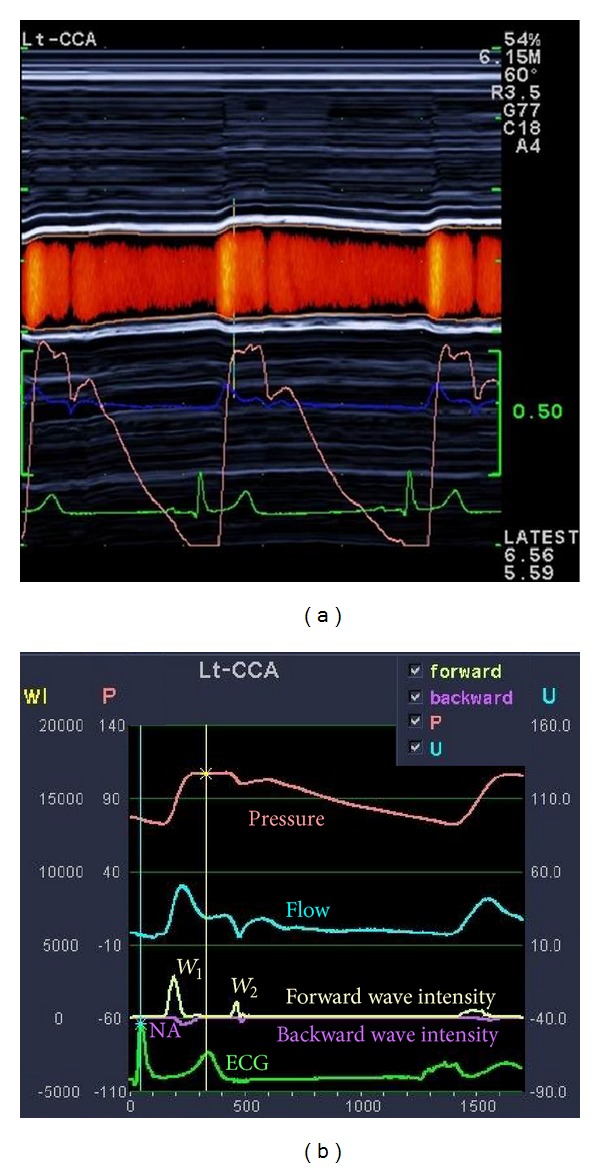
(a) Simultaneous measurement of CCA diameter changes (pink lines) creating the distension waveforms (analogous to a pressure waveform) and CCA blood flow velocity waveforms (dark blue) from echo tracking and color Doppler. (b) Representative view of results obtained from WIA. From top to bottom: distension (pressure) waveform, flow velocity waveform, wave intensity analysis (*W*_1_, *W*_2_, and NA), and ECG.

Eighteen young healthy nonsmoking men free of cardiovascular and metabolic diseases and not taking medications of any kind served as subjects for this investigation. All subjects provided written consent and this study was approved by the Institutional Review Board of Syracuse University. Participants fasted for ≥3 hours and refrained from vigorous exercise, caffeine, and alcohol the day of testing. Following 10 minutes of quiet supine rest, images of the left common carotid artery (CCA), just distal to the carotid bulb, were obtained using a 5.0–13.0 MHz linear-array probe (ProSound *α*7, Aloka, Tokyo, Japan). Echo-tracking was used to measure diameter changes within 1/16th of an ultrasound wavelength (0.013 mm) [23] creating a distension waveform almost identical to pressure waveforms (Figure 1(a)) [24]. Range gated color Doppler signals averaged along the Doppler beam were used to simultaneously measure flow velocity waveforms. An insonation angle of ≤60° was maintained for all measures. Sample volume was adjusted to encompass the entire vessel. At least 8 waveforms were ensemble-averaged to provide a representative average waveform. 

Wave intensity was calculated using time derivatives of blood pressure (*P*) and velocity (*U*), where wave intensity = (*dP*/*dt* × *dU*/*dt*); thus the area under the *dP*/*dt* × *dU*/*dt* curve represents the energy transfer of the wave (Figure 1(b)) [25]. WIA states that, if these wave fronts carry a positive rate of pressure change, they are referred to as compression waves. Conversely, if the wave front carries a negative rate of pressure change, they are referred to as expansion waves. It should be noted that “expansion” in this setting is an expression from fluid dynamics theory referring to “decreasing pressure” and not to be confused with “dilitation.” Using WIA, (1) *W*_1_ represents a forward compression wave produced during early systole that accelerates flow and increases pressure; (2) *W*_2_ represents a forward expansion/decompression wave that decelerates flow and reduces pressure; (3) the negative area (NA) between *W*_1_ and *W*_2_ is a backward travelling compression wave due to the sum of waves reflected from the periphery that decelerates flow but increases pressure [23, 25]. We additionally computed the WIA reflection index (RIx) as NA/*W*_1_. Arterial stiffness was assessed using a single-point pulse wave velocity (PWV) as previously described [26]:

(1) $$\begin{array}{c} \text{PWV}=\sqrt{\frac{\beta xP_{\mathrm{Min}}}{2\rho }},\;\text{where}\;\beta =\ln\frac{\left(P_{\mathrm{Max}}/P_{\mathrm{Min}}\right)}{\left[\left(D_{\mathrm{Max}}-D_{\mathrm{Min}}\right)/D_{\mathrm{Min}}\right]}. \end{array}$$

*P* and *D* correspond to pressure and diameter, respectively, and Max and Min refer to maximum (systolic) and minimum (diastolic) pressure values during the cardiac cycle, obtained from simultaneous assessment of carotid pressures from the contralateral CCA (calibrated against brachial mean and diastolic pressure assessed from an oscillometric cuff, described below). Blood density, *ρ*, is assumed constant and equal to 1050 kg/m^3^. 

**Figure 2:**
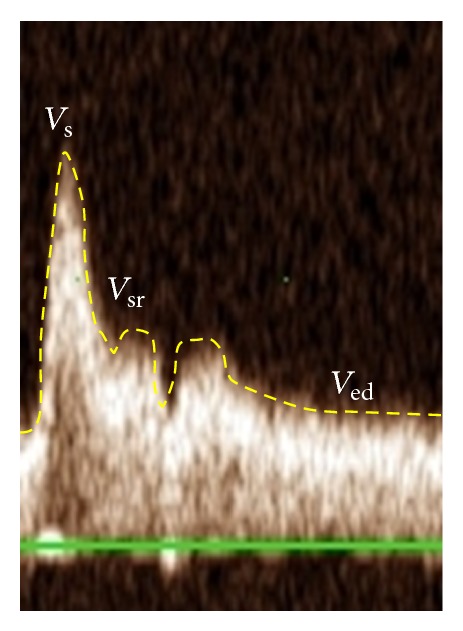
Sample CCA flow velocity waveform. *V*_*s*_: peak systolic flow velocity; *V*_*sr*⁡_: secondary rise in the CCA flow velocity waveform; *V*_ed_: velocity during end diastole.

Systolic peak (*V*_*s*_), late systolic peak/shoulder (*V*_*sr*⁡_), and diastolic (*V*_ed_) and mean blood velocities (*V*_*m*_) were measured using Doppler-ultrasound and calculated as follows: *V*_*m*_ = ∫*V*(*t*)*dt*/FT, where ∫*V*(*t*)*dt* is the velocity-time integral of the velocity waveform and FT is flow time. Carotid flow augmentation index (FAIx), as depicted in Figure 2, was calculated as (*V*_*sr*⁡_ − *V*_ed_)/(*V*_*s*_ − *V*_ed_). CCA pulsatility index (PIx) was calculated as (*V*_*s*_ − *V*_ed_)/*V*_mean_. CCA shear rate was calculated as 4 × (*V*_mean_/CCA mean diameter). Intima media thickness (IMT) was measured from the lumen-intima interface to the media-adventitia interface across a 5 mm region distal to the carotid bulb using semiautomated digital calipers. Middle cerebral artery (MCA) blood velocity was assessed via transcranial Doppler ultrasound using a 2 MHz probe (DWL Doppler Box-X, Compumedics, Germany). Velocity envelopes were obtained at a depth of 50–65 mm and the pulsatility index calculated as described above for the CCA. 

Simultaneous pressure waveforms were obtained from the contralateral CCA using applanation tonometry (SphygmoCor, AtCor Medical, Syndey, Australia). Following CCA measures, synthesized aortic (Ao) pressure waveforms were generated from radial artery pressure waves using a generalized transfer function. Carotid and aortic pressure waveforms were calibrated to brachial mean arterial pressure (MAP) and diastolic BP [27] obtained from simultaneous measures using an oscillometric cuff (Panasonic Ew3109, Secaucus NJ). Augmentation index (AIx) was calculated as the difference between the early and late systolic peaks of the pressure waveforms to the total pulse pressure and expressed as a percentage (*P*_2_ − *P*_1_/PP × 100). 

**Figure 3:**
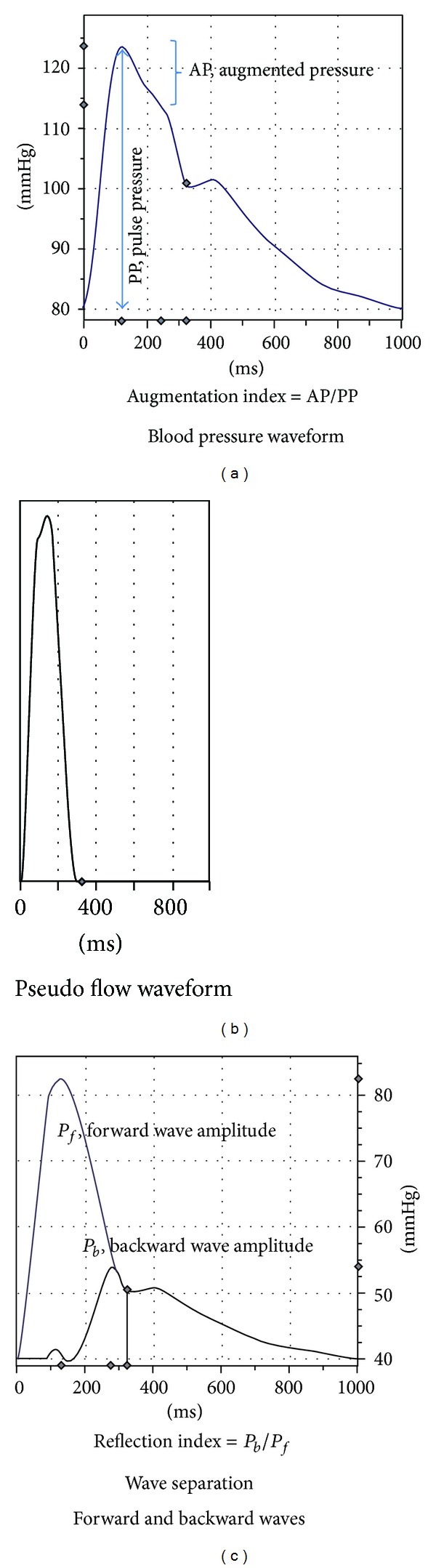
CCA pressure waveform (a) and pseudo flow waveform (b) used for wave separation analysis (c).

Using a modified pseudo average-flow waveform, carotid and aortic pressure waveforms were separated into forward (*P*_*f*_) and backwards/reflected (*P*_*b*_) components as previously described [28–30]. This rounded triangular flow waveform (Figure 3, generated by the SphygmoCor, AtCor Medical) assumes zero flow during diastole and is interpolated such that the base of the flow wave (triangle) corresponds to the upstroke of the pressure wave (pressure at waveform foot = time 0) and the incisura/dicrotic notch (i.e., aortic valve opening signifying the start of ejection and aortic valve closure signifying end ejection). Peak ejection is set at the inflection point (if augmented pressure > primary wave pressure) or peak pressure (if augmented pressure < primary wave pressure). More specifically, the inflection point is determined from the first negative zero crossing of the first derivative (inflection point occurs before peak pressure; pressure wave is transitioning from positive to negative). If a zero crossing is not present before peak pressure, peak ejection is determined from the positive zero crossing of the second derivative (inflection point occurs after peak pressure; pressure wave is transitioning from negative to positive) [31]. The forward and backward components of the pressure wave were constructed using the following equations:

(2) $$\begin{array}{c} \left(1\right)\;P_{f}\left(t\right)=0.5\left[P_{m}\left(t\right)+Zc\times Q_{m}\left(t\right)\right], \end{array}$$

(3) $$\begin{array}{c} \left(2\right)\;P_{b}\left(t\right)=0.5\left[P_{m}\left(t\right)-Zc\times Q_{m}\left(t\right)\right], \end{array}$$

where *P*_*m*_(*t*) is the measured time-varying pressure wave (aortic or carotid), *Q*_*m*_(*t*) is the approximated pseudo flow wave, *P*_*f*_ is the forward pressure component, and *P*_*b*_ is the backward pressure component. *P* and *Q* denote harmonics derived from Fourier decomposition of the pressure and flow signals into a series of sinusoidal harmonics with *Zc* being calculated by averaging the modulus of the 4th to the 7th harmonic of the input impedance (accounting for fluctuations due to wave reflections). Because calculation of *P*_*f*_ and *P*_*b*_ involves the product of flow and characteristic impedance (*Zc*), which itself has flow in the denominator, calibration of the flow waveform is not needed. Thus as seen in Figure 3, the flow scale is arbitrary/unit-less. Pulse transit time can be estimated from the time difference between the derived forward and reflected waves (maximum time lag determined from the highest cross-correlation of *P*_*b*_ and *P*_*f*_ normalized to same amplitude) and used to provide an estimate of aortic pulse wave velocity (PWV) [31]. The wave separation analysis (WSA) reflection index (RIx) was calculated as *P*_*b*_/*P*_*f*_. The aortic-carotid transmission index (TIx) was calculated as aortic RIx/carotid RIx and used to provide insight into aortic-carotid impedance matching. 

Normality of distribution was verified using Kolmogorov-Smirnov and Shapiro-Wilk tests. If a primary outcome variable was not normally distributed, it was log transformed for parametric analyses. Univariate associations of interest were analyzed using Pearson's correlation coefficients. All statistical analyses were performed using SPSS (version 20 IBM). 

## 3. Results

All results are presented as mean ± SEM. Participants were 22 ± 1 years of age with a body mass index of 24 ± 1 kg/m^2^. Resting brachial SBP and DBP were 122 ± 2 and 73 ± 1 mmHg, respectively. Mean values for carotid and aortic vascular-hemodynamic parameters are presented in Table 1 and Table 2, respectively. Values of RIx obtained from WIA were not normally distributed and were thus logarithmically transformed to allow for parametric statistical analyses. Primary correlates of FAIx were *W*_2_ (*r* = −0.52, *P* < 0.05), carotid WIA _log⁡_RIx (*r* = 0.56, *P* < 0.05), carotid pressure AIx (*r* = 0.60, *P* < 0.05), and carotid WSA RIx (*r* = 0.63, *P* < 0.05). FAIx was not directly associated with carotid IMT, carotid stiffness (measured as PWV), carotid shear, carotid pulsatility index, cerebral pulsatility index, and aortic stiffness (PWV) nor was it associated with aortic pressure AIx (*P* > 0.05). Correlates of the individual carotid velocity components that comprise FAIx are presented in Table 3. Of interest, *V*_*sr*⁡_ was inversely correlated with *W*_2_ (*P* < 0.05) and positively correlated with aortic AIx (*P* < 0.05) and aortic RIx (*P* < 0.05). Additional correlations of interest are as follows. TIx was associated with aortic PWV (*r* = 0.66, *P* < 0.05), CCA shear (*r* = 0.53, *P* < 0.05), CCA IMT (*r* = 0.56, *P* < 0.05), CCA pulsatility (*r* = 0.48, *P* < 0.05), and MCA pulsatility (*r* = 0.39, *P* = 0.06). CCA pulsatility was also associated with MCA pulsatility (*r* = 0.63, *P* < 0.05).

## 4. Discussion

Findings of this study support previous suggestions that FAIx is associated with measures of wave reflections. This is primarily due to attenuation of *V*_*s*_ (primary peak in early systole) by waves reflected from the cerebral circulation and augmentation of *V*_*sr*⁡_ (secondary peak in late systole) related to wave reflections arriving from the lower body/aorta. FAIx is also inversely associated with a forward travelling decompression wave of myocardial origin, suggesting a role for LV suction in affecting the carotid flow contour. This was primarily due to the inverse association between *W*_2_ and *V*_*sr*⁡_. Thus FAIx is a unique ventricular-vascular coupling parameter that reflects a complex interplay between LV properties (relaxation/suction), aortic-peripheral vascular properties (wave reflections from the descending aorta/lower body), and carotid-cerebral properties (wave reflections from the cerebral circulation). FAIx itself was not associated with CCA IMT or MCA PIx in young healthy adults. 

According to the pioneering work of Murgo et al., blood pressure waveforms may be characterized into any 1 of 4 categories based on their contour [32]. Type A and type C are waveforms of relevance to the current paper. Type Waveforms denote waveforms in which the late systolic shoulder is greater than pressure at a clearly defined inflection point yielding a positive AIx (usually >12%). Conversely type C waveforms are defined as those waveforms in which the late systolic pressure shoulder occurs after peak systolic pressure and is less than the primary wave pressure, yielding a negative AIx. Use of AIx as a measure solely attributable to pressure from wave reflections in young adults with type C waveforms has been challenged [33, 34]. In this setting, AIx “suffers from a tip-of-the-iceberg phenomenon [34]” in which only the pressure above the forward wave pressure is captured. Reflected wave pressure may be submerged along the falling edge of the forward pressure wave and/or may occur in diastole and in this setting AIx provides little insight into wave reflection magnitude as calculated values are negative (*P*_2_ < *P*_1_) [34]. Wave intensity analysis (WIA) and wave separation analysis (WSA) are distinct yet complimentary techniques that allow for a more parsimonious appraisal of pressure from wave reflections in young adults with type C waveforms [33, 35]. Using these techniques to calculate the reflection index, it was revealed that although carotid RIx was associated with FAIx, it was not associated with carotid late systolic flow augmentation *per se*. The cerebral circulation serves as a primary source of carotid wave reflections [36]. It is generally acknowledged that pressure from wave reflections augments incident wave pressure but attenuates antegrade flow [28, 37, 38]. Therefore, wave reflections of cerebral origin would be expected to attenuate flow in the CCA [39] manifesting as an inverse association between carotid RIx and *V*_*sr*⁡_ and this was not seen. CCA RIx was inversely associated with *V*_*s*_ suggesting that wave reflections are indeed important in affecting the carotid flow contour and FAIx. Our findings support computational multibranched fluid dynamics models put forth by Masuda et al. that have revealed that *V*_*s*_ is attenuated by arrival of a reflected pulse wave downstream of the CCA [5]. Owing to physical distance to the effective reflecting sites and/or speed of transit, wave reflections may arrive during midsystole rather than late systole, attenuating early systolic (*V*_*s*_) rather than late systolic (*V*_*sr*⁡_) flow velocity. 

There was a positive association between aortic wave reflections and CCA late systolic and diastolic flow (*V*_*sr*⁡_ and *V*_ed_) [39] and this is consistent with recent findings [6]. Masuda et al. also previously noted that *V*_*sr*⁡_ in the CCA occurred as a result of arrival of reflected waves from downstream of the thoracic aorta, increasing flow rate in the CCA in late systole [5]. Our correlative findings support results from this simulation study. Reflected waves in the aorta may enter the CCA as forward traveling compression waves serving to accelerate antegrade flow [40]. As such, the interface between aorta and carotid as related to cerebrovascular disease has recently received attention [3]. It has been suggested that disproportionate stiffening of the aorta as compared to the CCA affects regional impedance matching, reducing wave reflection at this junction and facilitating transmission of pulsatile flow into the cerebral circulation [3]. In support of this, we noted an association between aortic PWV and the transmission index. The transmission index was further associated with CCA shear, CCA IMT, CCA pulsatility, and MCA pulsatility. Thus alteration of wave reflection timing/magnitude in the aorta owing to increased PWV coupled with reduced wave reflection in the CCA is associated with greater transmission of flow pulsatility into the CCA and is further associated with regional shear rate, subclinical atherosclerosis/vascular wall damage (i.e., IMT), and MCA pulsatility. This may have important implications for cerebrovascular disease [3]. 

According to the present findings, the decompression/expansion wave was a direct correlate of late systolic flow augmentation and this is highly novel. *W*_2_ as measured herein is a forward travelling expansion wave created by myocardial shortening rate (LV relaxation) and inertial force of aortic blood flow (momentum) that causes a rapid fall in LV pressure [10, 25]. This creates a suction wave that serves to decelerate flow [41]. Waves in the systemic circulation with a pulling effect have been known to exist for quite some time [42]. This suction wave has been shown to be an important correlate/moderator of late systolic/early diastolic flow in the coronary [12, 43], aorta [8, 10], and femoral arteries [14]. Most notably, this suction wave has been implicated as a factor contributing to aortic flow reversal and valve closure [8, 9]. It is interesting to note that retrograde flow in the aorta has recently been shown to be associated with late systolic/early diastolic antegrade flow in the CCA [6]. Our findings extend and link these observations suggesting for the first time that the expansion/suction wave is also associated with late systolic flow in the CCA.

Overall findings suggest a complex interplay between LV, aortic, and carotid hemodynamics in affecting the contour of the carotid flow waveform in young healthy adults. Based on current observations, late systolic flow augmentation in the carotid artery may be due to the summative effect of increased forward wave pressure from aortic origin arriving in midlate systole (reflected waves entering the carotid artery as forward waves causing flow acceleration, i.e., “increased push from behind”) and reduced suction (“decreased pull from behind”). This “push-pull” balance may be altered with aging or in the presence of hypertension owing to increased aortic stiffness and/or reduced LV function. More research will be needed to examine these hemodynamic interactions with aging and disease. 

Limitations to this study must be acknowledged. This is a purely correlative study in a small rather homogenous group of participants. Correlation does not imply causation. As such findings should be viewed as hypothesis generating rather than demonstrative. We believe that these findings, however small, do fill an important void in the literature as studies to date that have examined hemodynamic correlates of carotid flow have done so in middle-age/older adults with predominantly type A and type B waveforms. Many of the parameters were derived from the same pressure and flow waveforms; thus there is potential that existence of relations is due to methodological colinearity. CCA pressure, and subsequent measures derived from the CCA pressure waveform, was not measured in the same artery as that used to assess FAIx and measures of target organ damage (IMT and MCA PI). Impedance may differ between left and right CCA owing to anatomical differences between arteries. The left CCA directly connects to the aortic arch, while the right CCA indirectly connects to the ascending aorta via the innominate/brachiocephalic artery. Indeed, flow velocity is higher in the left CCA in young adults compared to the right CCA and may explain the slightly greater IMT in left CCA in later life [44]. It should be noted that hemodynamic variables derived from WIA were measured in the same CCA as that used to assess FAIx and measures of target organ damage and results obtained from WIA support results from WSA. 

In conclusion, FAIx is a complex parameter that reflects the integrated effects of LV, carotid, and aortic hemodynamics on specific CCA flow components across the cardiac cycle. Late systolic flow augmentation in the CCA is associated with both increased expansion wave magnitude (suction from the LV) and increased pressure from wave reflections. 

## Figures and Tables

**Table 1:** Carotid hemodynamic values (mean ± SEM).

Variable	Value
Systolic blood pressure, mmHg	116 ± 2
Diastolic blood pressure, mmHg	73 ± 2
Heart rate, bpm	60 ± 2
*V*_*s*_, cm/s	108.4 ± 4.9
*V*_*sr*⁡_, cm/s	45.1 ± 2.2
*V*_ed_, cm/s	24.0 ± 1.1
Flow augmentation index, %	23.9 ± 1.8
*W*_1_, mmHg·m·sec^−3^	9.4 ± 0.8
*W*_2_, mmHg·m·sec^−3^	3.2 ± 0.5
Negative area, mmHg·m·sec^−2^	−47.9 ± 12.9
WIA _log_reflection index, %	0.67 ± 0.1
Pulse wave velocity, m/s	4.2 ± 0.2
Pressure augmentation index, %	−17 ± 3
Forward wave pressure, mmHg	41 ± 2
Backward wave pressure, mmHg	16 ± 1
WSA reflection index, %	39 ± 2
Pulsatility index	2.1 ± 0.1
Shear rate, s^−1^	276.6 ± 12.9
Intima media thickness, mm	0.44 ± 0.02
MCA pulsatility index	0.55 ± 0.01

CCA: common carotid artery; MCA: middle cerebral artery; WIA: wave intensity analysis; WSA: wave separation analysis.

**Table 2:** Aortic hemodynamic values (mean ± SEM).

Variable	Value
Systolic blood pressure, mmHg	111 ± 2
Diastolic blood pressure, mmHg	74 ± 1
Pulse wave velocity, m/s	7.5 ± 0.2
Pressure augmentation index, %	−3 ± 2
Forward wave pressure, mmHg	34 ± 2
Backward wave pressure, mmHg	14 ± 1
WSA reflection index, %	41 ± 2
Transmission index	1.04 ± 0.06

WSA: wave separation analysis; aU: arbitrary units.

**Table 3:** Hemodynamic correlates of the carotid flow waveform.

Variable	*V*_*s*_	*V*_*sr*⁡⁡_	*V*_ed⁡_
*W*_2_	−0.04	**−0.43**	0.25
CCA WIA _log_RIx	**−0.42**	−0.16	0.16
CCA PWV	**−0.60**	−0.20	−0.08
CCA Aix	**−0.44**	−0.04	0.20
CCA WSA RIx	**−0.57**	−0.10	0.03
Ao AIx	**0.49**	**0.47**	**0.45**
Ao PWV	0.33	0.32	**0.43**
Ao WSA RIx	**0.43**	**0.52**	**0.47**
Ao-CCA TIx	**0.70**	**0.44**	**0.58**
CCA PIx	**0.71**	0.25	**−0.45**
CCA Shear	**0.62**	**0.42**	**0.76**
CCA IMT	0.32	−0.08	0.25
MCA PIx	**0.53**	0.01	−0.12

Bold significant association (*P* < 0.05).

## Abbreviations

FAIx: Flow augmentation index
LV: Left ventricle
WIA: Wave intensity analysis
WSA: Wave separation analysis
CCA: Common carotid artery
MCA: Middle cerebral artery
AIx: Augmentation index (pressure)
RIx: Reflection index
Pix: Pulsatility index
IMT: Intima media thickness
TIx: Transmission index.
